# Evaluation of the Antibacterial and Antioxidant Properties of Chemical Constituents of the Roots of *Woodfordia uniflora*: An Integrated Approach of Experimental and Computational Study

**DOI:** 10.1155/bri/1322756

**Published:** 2024-12-03

**Authors:** Bihon Abera, Negera Abdissa, Milkyas Endale, Yadessa Melaku, Kebede Shenkute, Urgessa Ensermu, Mo Hunsen, Daniel Rentsch, Rajalakshmanan Eswaramoorthy

**Affiliations:** ^1^Department of Applied Chemistry, School of Applied Natural Science, Adama Science and Technology University, P.O. Box 1888, Adama, Ethiopia; ^2^Traditional and Modern Medicine Research and Development Directorate, Armauer Hansen Research Institute, P.O. Box 1005, Addis Ababa, Ethiopia; ^3^Department of Applied Biology, School of Applied Natural Science, Adama Science and Technology University, P.O. Box 1888, Adama, Ethiopia; ^4^Department of Chemistry, Kenyon College, Gambier, Ohio 43022, USA; ^5^Laboratory for Functional Polymers, Empa, Swiss Federal Laboratories for Materials Science and Technology, Überlandstrasse 129, Dübendorf 8600, Switzerland; ^6^Department of Biomaterials, Saveetha Dental College and Hospitals, Saveetha Institute of Medical and Technical Science, Saveetha University, Chennai, India

**Keywords:** antibacterial, antioxidant, binding affinity, molecular docking, *Woodfordia uniflora*

## Abstract

*Woodfordia uniflora* is a medicinal plant used for the treatment of malaria, toothache, and stomach problems. The root parts of the plant are also used for healing liver disorders. Silica gel chromatography separation of CH_2_Cl_2_/MeOH (1:1) and MeOH extracts of roots of *W. uniflora* result in the isolation of three compounds, namely, bergenin (**1**), *β*-sitosterol (**2**), and epigallocatechin 3-gallate (**3**), reported herein for the first time from the plant. The structure of the isolated compounds was elucidated using NMR (1D and 2D) techniques. Disk diffusion and DPPH assay were used to evaluate the antibacterial and antioxidant activities, respectively. Molecular docking was done by the AutoDock Vina 4.2 program. The pharmacokinetics and toxicity profile of compounds were predicted by Swiss ADME and Pro Tox II online servers. GC-MS analysis roots of *W. uniflora* result in the identification of five compounds, of which palmitic acid (34.9%) was the major constituent. The antibacterial activity result indicated that the oil extract had promising activity against *P. aeruginosa*, *E. coli*, *S. pyogenes*, and *S. aureus* with IZ of 14.3 ± 0.81, 15.0 ± 0.0, 15.6 ± 0.47, and 17.6 ± 0.47 mm, respectively, at 5 mg/mL, compared to ciprofloxacin (1Z 27–30.0 ± 0.0 mm) at 30 *μ*g/mL. MeOH and CH_2_Cl_2_/MeOH (1:1) extract showed inhibition against *E. coli* (IZ of 13.6 ± 0.47 mm) and *P. aeruginosa* (IZ of 10.0 ± 0.0 mm), respectively, at 200 mg/mL. Bergenin (**1**) and *β*-sitosterol (**2**) also displayed maximum inhibition of *E. coil* (IZ of 11.6 ± 0.47) and *S. aureus* (11.0 ± 0.0 mm), respectively, at 5 mg/mL. The antioxidant activity results showed that CH_2_Cl_2_/MeOH (1:1) and MeOH extracts, bergenin (**1**), and compound **3** displayed potent scavenging DPPH radical with a percentage of inhibition of 76.8 ± 0.12, 77.8 ± 0.08, 71.4 ± 0.08, and 91.2 ± 0.16, respectively, compared to ascorbic acid (93.2% ± 0.04%) at 100 *μ*g/mL. The molecular docking analysis showed that all compounds (**1**–**3)** exhibited minimum binding energy toward PDB ID: 1HD2 (−5.2 to −6.3 kcal/mol), compared to ascorbic acid (−5.6 kcal/mol), and toward PDB ID: 1DNU (−8.0 to −10.7 kcal/mol) receptors, compared to ascorbic acid (−5.7 kcal/mol). Toward the PDB ID: 4FM9 receptor, *β*-sitosterol (**2**) and compound **3** exhibited the best binding free energy of −9.1 and −9.8 kcal·mol, respectively, compared to vosaroxin (−7.8 kcal/mol). The drug-likeness analysis result indicated that bergenin (**1**) and *β*-sitosterol (**2**) obeyed four and five criteria of Lipinski's rule, respectively, and are more likely to be administered orally. The *in silico* toxicity analysis showed none of the compounds would be cytotoxic, mutagenic, or hepatotoxic. The in vitro antioxidant and antibacterial results supported by in silico analysis demonstrated that the roots of *W. uniflora* have the potential to be therapeutic agents for bacterial infections and free radical–inducing diseases.

## 1. Introduction

Infectious diseases have been a serious health problem and found to be one of the main threats to world health. According to the World Health Organization, infectious diseases, which account for about 50% of all fatalities in tropical nations, are a major cause of morbidity and mortality [1]. The existence of multidrug-resistant microorganisms is increasingly limiting the effectiveness of currently available drugs [2]. Despite the availability of a variety of antibiotic medications, antibiotic drug resistance has continued to negative impact the public health sector [3]. The prolonged bacterial infections related to high illness and fatalities are generally established through the production of highly reactive oxygen species (ROS) and related oxidative stress [3, 4]. Oxidative stress is caused by an imbalance between free radical production and natural antioxidants. It is associated with damage to a wide range of molecular species including lipids, proteins, and nucleic acids, leading to cellular dysfunctions and cell death via necrosis or apoptosis [5]. Therefore, scavenging of reactive free radicals is an important mechanism to prevent oxidative stress-inducing diseases. Importantly, natural compounds possess a range of potent biological properties, including antibacterial, antitumor, anti-inflammatory, and antioxidant properties [6, 7]. Antioxidant compounds have gained attention for their ability to reduce the actions of free radicals either by metal chelating, and inhibition of enzyme activities, or scavenging free radicals [8]. Bioefficient natural compounds are an important source of new antioxidants and antibacterial agents [9]. Natural product chemists have been working to purify and characterize the herbal formulations and decoctions utilized by indigenous communities around the world to find new bioactive compounds [10]. The therapeutic values of different medicinal plants are associated with the presence of a wide range of chemical molecules. Identifying and predicting the pharmacological basis of the activity of traditional medicinal plants are important for the foundation of modernizing drugs [11]. The conventional method for identifying biologically active compounds from natural products is expensive and time-consuming [12]. Computer-aided drug discovery methods have become more effective in finding drugs derived from phytochemicals found in a wide range of medicinal plants. Additionally, they have been applied to in silico predictions of toxicological, pharmacological, and pharmacokinetic properties [13]. Molecular docking mostly attempts to predict the binding orientations of drug candidates in response to a particular target protein to achieve a specific drug design and discover a novel drug [14].

**Figure 1:**
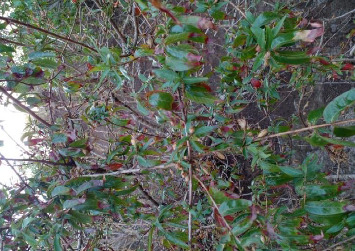
*Woodfordia uniflora* (Photo taken by Bihon Abera, in 2022).

The genus *Woodfordia* (Lythraceae) comprises two well-known morphologically similar species, namely, *Woodfordia fruticosa* and *Woodfordia uniflora*. *W. fruticosa* is widely found in Asia, while *W. uniflora* (Figure 1) is extended in the Middle East and East Africa [15]. Literature reviews documented on *W. uniflora* indicated that the plant has a long history for the medicinal value of rheumatism, bowel complaints, hematuria [16], alexiteric, dysentery, and toothache [17, 18]. In the Oman region, *W. uniflora* has been used as a sedative and medication for skin ailments [19]. In Ethiopia, the fresh root portion of *W. uniflora* is also used to heal liver disorders [20]. Limited studies on various parts of *Woodfordia* species reported the presence of phenolic compounds, mainly hydrolysable tannins and flavonoids [21, 22]. Motivated by the wide spectrum of its traditional uses and limited prior phytochemical studies, the present study herein presents the isolation of chemical constituents, biological activities, and molecular docking analysis of CH_2_Cl_2_/MeOH (1:1) and MeOH root extracts of *W. uniflora*.

## 2. Materials and Methods

### 2.1. General Experimental Procedure

^1^H and ^13^C NMR spectral data were analyzed on a Bruker Avance 400 MHz spectrometer. CDCl_3_, DMSO − *d*_6_, and CD_3_OD were used as solvents for NMR analysis. A precoated aluminum plate (silica gel 60 F_254_) was used for TLC analysis and compounds on TLC were identified with UV lamp (at 254 and 365 nm) and vanillin/H_2_SO_4_. Column chromatography was used for the isolation of compounds (silica gel mesh size of 60–120 mm as stationary phase). Essential oil analysis was performed on gas chromatography-mass spectrometry (GC-MS) (Agilent 7890B) technology. P9-VWR double-beam UV–Vis spectrophotometer was used during absorbance measurement at 517 nm. A digital melting point apparatus was used for determining the melting points of solid compounds.

### 2.2. Plant Material Collection

The roots of *W. uniflora* were collected in April 2022 from Lalibela Town, North Wollo Zone, Amhara Regional State, Ethiopia, located about 710 km north of the capital, Addis Ababa. The plant was authenticated by Mr. Melaku Wondafrash, and a voucher code for specimen BA002 was assigned and deposited at the National Herbarium, Department of Biology, Addis Ababa University, Addis Ababa, Ethiopia.

### 2.3. Extraction and Isolation of Compounds

Powdered roots of *W. uniflora* (700 g) were soaked with 4 L of CH_2_Cl_2_/MeOH (1:1) for 72 h, and the extract was filtered. The process was repeated twice, and the combined extract was concentrated using a rotary evaporator at 40°C to afford 38 g (5.4%) extract. The marc was soaked with methanol (3 L) for 72 h, filtered, and concentrated, and the process was repeated twice to afford 26 g (3.7%). Preliminary TLC profiles of the crude extracts were analyzed, and both CH_2_Cl_2_/MeOH (1:1) and MeOH extracts exhibited similar spots under a UV lamp. Afterward, both crude extracts were combined, dissolved with methanol, and poured into a 1000-mL separatory funnel containing 400 mL of distilled water and chloroform (400 mL) was added and shaken continuously at room temperature. A white layer was formed between the organic (chloroform) and the aqueous phase. A white layer fraction was collected, and the process was repeated twice. A white layer and chloroform soluble fraction were concentrated to afford 3.5 and 4 g yields, respectively. The white layer fraction showed one major spot under the UV_254_ lamp (90% EtOAc in *n*-hexane as eluent) afforded compound **1** (3.5 g, 0.5% w/w).

Chloroform soluble fraction (4 g) was adsorbed with 4 g of silica gel and subjected to chromatographic separation (silica gel 110 g, 60–120 mesh size as stationary phase) and eluted with increasing gradient of EtOAc in *n*-hexane. A total of 86 fractions (each 100 mL) were collected. The TLC profile of each fraction was detected under a UV lamp (254 and 365 nm) and visualized after spraying with vanillin/H_2_SO_4_. Fractions 18–21 (eluted with 20% EtOAc in *n*-hexane) were combined and washed further with *n*-hexane to afford compound **2** (45 mg). Fractions 40 and 41 were combined and purified by column chromatography increasing the gradient of EtOAc in *n*-hexane as eluent to collect a total of 84 fractions (each 10 mL). Subfractions 78 and 79 showed a single spot under UV_245_ nm to afford compound **3** (26 mg).

### 2.4. Oil Extraction and GC-MS Analysis

Hydrodistillation extraction and GC-MS analysis of the essential oil constituents of the root of *W. uniflora* were carried out following the standard procedures as described in the literature [23]. The powdered root of *W. uniflora* (60 g) was mixed with distilled water (300 mL) in a round-bottom flask and connected to a Clevenger apparatus and then heated for 3 h at 70°C. The organic phase was separated using a separatory funnel and dried by anhydrous Na_2_SO_4_ as a water-absorbent agent. Finally, the obtained essential oil was measured and kept at −4°C in the refrigerator for further analysis.

GC-MS analysis of the essential oil was established using an Agilent 7890B gas chromatograph with an Agilent 5977B GC/MSB mass spectrometer equipped with an HP-5MS capillary column (30 m × 0.25 mm, 0.25 µm film thickness). The GC condition was adjusted as follows: The oven temperature was adjusted at 70°C for the first 2 minutes and gradually increased by 5°C per minute until it reached 300°C. After that, the temperature was adjusted to remain constant for the next 2 minutes; the injector temperature and volume were kept at 250°C and 1 μL, respectively, with a split ratio of 1:20, and the medium of transport was helium. Condition for MS: Ion spring temperature was 230°C, scan range of 30–500 Amu, and EI ionization mode, 70 eV. The relative percentages of the identified compounds were calculated based on chromatograms obtained from the GC/FID/MS system [24].

### 2.5. Antibacterial Activity

The antibacterial potentials of CH_2_Cl_2_/MeOH (1:1) and MeOH root extracts, oil extract, and isolated compounds (**1**–**3**) were investigated against *S. pyogenes*, *E. coli*, *S. aureus*, and *P. aeruginosa* bacterial strains. Disk diffusion technique was employed based on standard guidelines described in the reported literature [25]. In brief, a few colonies (3–5) of bacterial strains with similar morphologies were properly introduced into a liquid medium (saline solution) using a sterilized inoculating loop. The turbidity of the suspension was adjusted to 0.5 Mc Farland barium sulfate standards (10^8^ CFU/mL). The experiment was carried out at twofold serial solutions of 200, 100, and 50 mg/mL of CH_2_Cl_2_/MeOH (1:1) and MeOH crude extracts, 5, 2.5, and 1.25 mg/mL of isolated compounds (**1**–**3**) using DMSO as the diluting agent. Bacterial suspensions were applied onto a petri plate containing MHA. Sterile Whatman paper disks (6 mm) were applied on the surface of the inoculated MHA, and about 20 µL each test sample was applied onto the disks. Ciprofloxacin (30 μg/mL) was used as the reference drug.

### 2.6. Radical Scavenging Activity

The radical scavenging activities of CH_2_Cl_2_/MeOH (1:1) and MeOH extracts, oil extract, and isolated compounds (**1**–**3**) were evaluated using DPPH assay as described by Sasikumar, Erba, and Egigu [26] using ascorbic acid as standard. Twofold serial dilution solutions of 100, 50, 25, 12.5, and 6.25 μg/mL were prepared from 1 mg/mL stock solutions. 4 mL of DPPH (dissolved in methanol) was added to 1 mL of each serially prepared sample and kept for 30 min in the dark. The absorbance was measured using a UV–Vis spectrophotometer at 517 nm.

The percentage of inhibition of DPPH radical was calculated as follows:

(1) $$\begin{array}{c} \%\text{ inhibition}=\frac{\left(A_{\text{control}-}A_{\text{sample}}\right)}{A_{\text{control}}}\times 100, \end{array}$$

where *A*_control_ is the absorbance of DPPH and *A*_sample_ is the absorbance of the sample with DPPH solution. The inhibitory concentration, the concentration needed to scavenge 50% of DPPH radical (IC_50_), was calculated from the Prism dose–response curve (GraphPad, Prism version 4.0 for Windows, GraphPad Software, San Diego, CA, USA) obtained by plotting the percentage of inhibition versus the concentrations [27].

### 2.7. Molecular Docking Analysis

The assessment of the orientation and binding affinity of the ligands (compounds **1**–**3** and ascorbic acid) toward human myeloperoxidase (MPO) (PDB ID 1DNU), human peroxiredoxin 5 (Prdx5) (PDB ID: 1HD2), and topoisomerase II*α* (Topo II*α*) (PDB ID: 4FM9) receptor were carried out based on standard procedures as described by Uttu and co-authors [28]. AutoDock Vina tools were employed for molecular docking analysis.

#### 2.7.1. Protein and Ligand Preparation

The 3D crystal structures of the selected target proteins (PDB ID: 1HD2, PDB ID: 1DNU, and PDB ID: 4FM9) were downloaded from the RCSB Protein Databank (RCSB PDB: Homepage). The protein preparation was done using AutoDock 4.2.6 (MGL tools 1.5.6) following the standard protocol [28] by removing the cocrystallized ligand, water molecules, and cofactors. Polar hydrogens and Kollman charges were added to the protein, and a pdbqt format file was generated using the AutoDock Tools 1.5.6 software. The chemical structures of the studied compounds (**1**–**3**) were drawn using ChemOffice tool (Chem Draw 16.0) assigned with proper 2D orientation, and the energy of each molecule was minimized using ChemBio3D. The energy-minimized ligand molecules were saved in pdbqt format using the AutoDock Vina tool to carry out the docking simulation [29].

#### 2.7.2. Docking Simulations

A grid box size of 54 × 54 × 54 Å pointing in *x*, y, and *z* directions with a grid point spacing of 0.375 Å was established using the graphical user interface program AutoDock 4.2.6. The AutoDock Vina searched for the best-docked conformation between isolated compounds and target proteins. Nine different conformations were generated for each ligand in the docking process using the Lamarckian genetic algorithm (LGA) program. The conformations with the most favorable (least) binding free energy were selected for analyzing the interactions between the target receptor and ligands by Discovery Studio visualizer and PyMOL. The ligands are represented in different colors, H-bonds, and the interacting residues are represented in stick model representation [30, 31].

### 2.8. In Silico Pharmacokinetics Analysis of Isolated Compounds

The pharmacokinetics properties of compounds (**1**–**3**) were assessed to estimate their potential to be a drug candidate. This estimation was carried out based on the standard protocol adopted by the Lipinski rule [32]. Pro Tox II was employed to predict the isolated compounds' toxicities, toxicological endpoints, and LD50 (**1**–**3**) [33].

## 3. Results and Discussion

### 3.1. Structural Elucidation of Isolated Compounds

Compound **1** was obtained as a white powder with a melting point ranging from 238 to 242°C. Its TLC profile showed a single spot with *R*_*f*_ value of 0.51 (EtOAc: *n*-hexane (9:1) as eluent). Its ^1^H NMR (400 MHz, in CD_3_OD, Table 1, Supporting Information 1A) displayed a signal at *δ* 7.05 (1H, s) attributed to one aromatic methine proton. The signals observed at *δ* 4.94 (1H, d, *J* = 10.6), 3.68 (1H, m), 3.44 (1H, dd, *J* = 9.5, 8.7), 3.81 (1H, dd, *J* = 9.5, 8.7), and 4.05 (*J* = 10.4, 9.5) belong to sp^3^ oxygenated methine protons. The peak at *δ* 3.89 (3H, s) was attributed to the methoxy proton. The peaks at *δ* 3.72 (1H, m)) and 4.02 (1H, m) belong to diastereotopic oxygenated methylene protons. The ^13^C and DEPT-135 (Table 1, Supporting Information 1B and 1C) of compound **1** showed a total of 14 well-resolved carbons of which the peaks at *δ* 152.2, 149.6, and 142.2 belong to oxygenated quaternary aromatic carbons and the peak at *δ* 165.7 belongs to an ester carbonyl, whereas the peaks at *δ* 119.3 and 117.2 suggest the presence of two sp^2^ nonoxygenated quaternary carbons. The presence of sp^2^ methine was evident at *δ* 110.8 along with five sp^3^ oxygenated methine carbons at *δ* 71.9, 74.0, 75.5, 81.3, and 82.9. The peaks at *δ* 62.6 and 60.8 belong to oxygenated methylene and methoxy carbons, respectively.

**Figure 2:**
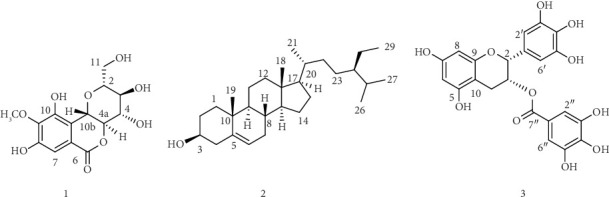
Compounds (**1**–**3**) isolated from the roots of *W. uniflora*.

Its HSQC spectrum (Table 1, Supporting Information 1D) indicated ^1^J direct correlations between signals at *δ* 4.94, 3.68, 3.44, 3.81, 4.05, 3.89, and 7.05 with carbon signals at *δ* 74.0, 82.9, 71.9, 75.5, 81.3, 60.8, and 110.8, respectively. The diastereotopic methylene protons at *δ* 3.72 and 4.02 displayed a ^1^J correlation with carbon at *δ* 62.6. The ^1^H-^1^H COSY spectrum (Table 1, Supporting Information 1E) of compound **1** revealed the correlations between protons appearing at *δ* 3.44 (H-3) with protons observed at *δ* 3.68 (H-2) and 3.81 (H-4) and vice versa. The COSY spectrum also showed the correlations of a proton signal displayed at *δ* 4.05 (H-4a) with a proton observed at *δ* 4.94 (H-10b) and vice versa. The COSY spectrum also confirms cross-coupling of the diastereotopic methylene protons displayed at *δ* 3.72 and 4.02. Its HMBC spectrum (Table 1, Supporting Information 1F) revealed the correlation of the methine proton displayed at *δ* 7.05 with carbon signals at *δ* 119.3 (C-6), 117.2 (C-10a), 142.2 (C-9), 149.6 (C-10), and 152.2 (C-8). Proton signal displayed at *δ* 4.94 (H-10b) showed HMBC correlation with oxygenated methine carbons resonated at *δ* 82.9 (C-2), 75.5 (C-3), 72.0 (C-4), and 81.3 (C-4a) and an ester carbonyl at *δ* 165.7 (C-6a). Proton signal at *δ* 4.02 (H-4a) also displayed HMBC correlation with carbon signals at *δ* 71.9 (C-4), 75.5 (C-3′), and 74.0 (C-10b). The above spectral data are in good agreement with data reported for bergenin (**1**) which was previously reported from *Peltophorum dubium*, *Flueggea leucopyrus*, and *Flueggea virosa* species [34–37]. Bergenin (**1**) (Figure 2) is a cytoprotective and antioxidative polyphenol found in many medicinal plants with a wide spectrum of activities such as hepatoprotective [38], anti-inflammatory [39], immunomodulatory [40], antitumor [41], antiviral [42], and antifungal [43] properties.

Compound **2** was obtained as a white crystalline solid with a melting point ranging from 135°C to 138°C (*R*_*f*_ value of 0.52 using EtOAc: *n*-hexane, 2:8 as mobile phase). Its ^1^H NMR (400 MHz, Table 2, Supporting Information 2A) exhibited two set of singlet protons integrated for three protons each at *δ* 0.70 (H-18) and 1.01 (H-19) suggesting the presence of two angular methyl carbons and signals displayed at *δ* 5.36 (1H, t), 3.53 (1H, m), *δ* 2.32 (1H, d), and 2.02 (1H, m) belong to an olefinic proton (H-6), sp^3^ methine proton bearing hydroxyl group (H-3), and methylene protons of H-4 and H-7, respectively, indicating the characteristic signals for β-sitosterol skeleton [45].

^13^C NMR and DEPT-135 spectra (Table 2, Supporting Information 2B and 2C) of compound **2** showed a total of 29 well-resolved peaks of which six methyls, nine methines, eleven methylenes, and three quaternary carbons were identified. The peaks at *δ* 140.7 and 121.7 belong to sp^2^ quaternary carbon and sp^2^ methine (C-5 and C-6), whereas the peak observed at *δ* 71.7 belong to sp^3^ methine-bearing hydroxyl group (C-3). Two angular methyl carbons appeared at *δ* 11.8 (C-19) and 19.3 (C-18). Based on the above spectral data, compound **2** was identified as *β-*sitosterol (**2**) (Figure 2) previously reported from *Baccaurea macrocarpa* species [44].

Compound **3** was isolated as yellowish gum with an Rf value of 0.6 using 80% EtOAc in *n*-hexane as eluent. ^1^H NMR spectrum (400 MHz, in DMSO − *d*_6_, Table 3, Supporting Information 3A) showed the presence of diastereotopic methylene protons shown at *δ* 2.65 (H4a, 1H, m) and 2.94 (H4b, 1H, m) attached to sp^3^ oxygenated methine at *δ* 4.94 (H-3, 1H, m) and 5.35 (1H, H-2). In total, six aromatic protons were observed of which two of them appeared with AB spin pattern at *δ* 5.82 (H, d, *J* = 2) and 5.93 (1H, d, *J* = 2) (Ring A), whereas the remaining four appeared as AA'BB' spin system at *δ* 6.40 (2H, s) and 6.80 (2H, s) (Ring B). The above ^1^H NMR spectra data suggest a catechin skeleton [47]. The signals observed at *δ* 8.38 (brs), 8.80 (brs), 9.17 (brs), and 9.44 (brs) belong to phenolic hydroxyl groups. The ^13^C NMR and DEPT-135 spectra (Table 3, Supporting Information 3B and 3C) of compound **3** revealed the presence of aliphatic methylene carbon displayed at *δ* 26.1, sp^3^ oxymethine at *δ* 68.5 and 76.9, AA′BB′ spin pattern aromatic methines at *δ* 105.9 and 109.0, and *α*, *β*-unsaturated ester group at *δ* 165.7. A total of eight sp^2^ quaternary carbons were observed at *δ* 119.7, 129.0 132.7, 138.9, 145.8, 146.0, 156.0, and 156.9. Two sp^2^ oxygenated symmetry carbons were displayed at *δ* 146.0 (C-3′/5) and *δ* 145.8 (C-3″/5″). The presence of two sp^2^ oxygenated aromatic quaternary carbons at *δ* 132.7 (C-4′) and *δ* 139.0 (C-4″) suggests 1,3-di-oxygenated a pattern at Ring B and galloyl moiety, respectively. The weak signals displayed at *δ* 48.9 and 60.3 are impurities and not a part of the compound. Based on the above aforementioned spectral data and comparison with published literature, compound **3** was identified as epigallocatechin 3-gallate (Figure 2), reported from various medicinal plants [46, 48, 49]. This compound has been reported from green tea with proven biological activity including as discrepant in cancer, bone regeneration, and nervous system [50].

### 3.2. Oil Profile and GC-MS Analysis

Medicinal plants are a rich source of natural oils such as essential oils and fixed oils used traditionally for nutritive as well as medicinal value [51]. Essential oil chemical constituents have a low molecular weight (below 300) and are rich in terpenes, sesquiterpenes esters, aldehydes, and phenols [52]. The breakdown of phospholipids and oily substances during metabolic change also produces nonterpenoid aliphatic hydrocarbon found in essential oil. Fixed oils are the portion of a nonvolatile oil that mostly consists of plant-derived plant materials such as fats, resins, and waxes [53]. In the present study, hydrodistillation of air-dried powdered roots of *W. uniflora* (60 g) afforded 8 mg (0.013% w/w yields). The GC-MS analysis results indicated that a total of five compounds were identified, comprising 99.97% of a total oil constituent (Table 4). Palmitic acid was the major component of the roots of *Woodfordia unifora* which comprises about 34.9% of the total oil content. The chemical profile of the extracted oils confirmed that essential oils, and fixed oils, and long-chain aliphatic hydrocarbons were the constituents of the oil part of the roots of the plant. Cyclopropene, 2,3-dimethyl-3-phenyl, a phenyl derivative of hemiterpenes, and (1R, 7S, E)-7-isopropyl-4, 10-dimethylenecyclodec-5-enol, which accounts about 34.18% of the total oil component, are the representative of essential oils found in the roots of *W. uniflora*. Palmitic acid, tricosane, and eicosane, which account for about 65.79% of the total oil component, were nonterpenoid aliphatic hydrocarbons representative of the fixed oil component of the root of the plant.

### 3.3. Biological Activities

#### 3.3.1. Antibacterial Activity

Evaluation of the antibacterial potentials of crude extracts, oil extract, and isolated compounds (**1**–**3**) was conducted against Gram-positive (*S. aureus* and *S. pyogenes*) and Gram-negative (*E. coli* and *P. aeruginosa*) bacteria strains following agar disk diffusion assay protocol [25]. The results of the antibacterial activities of the tested samples are presented in Tables 5 and 6. All tested samples showed low-to-moderate activity compared to ciprofloxacin. As shown in Table 5, MeOH extract exhibited antibacterial activity against all tested pathogens, and the maximum activity was recorded against with inhibition zone (IZ) of 13.6 ± 0.47 mm at 200 mg/mL, compared to ciprofloxacin (IZ of 27 ± 0.0 mm) at 30 *μ*g/mL. Cl_2_CH_2_/MeOH extract also displayed antibacterial activity against *E. coli* and *P. aeruginosa* with maximum IZ of 11.6 ± 0.81 and 10.0 mm, respectively, but did not show activity against *S. aureus* and *S. pyogenes* at the same concentration (Table 5).

As given in Table 6, bergenin (**1**) and compound **3** exhibited maximum antibacterial activity against *E. coli* with IZ of 11.6 ± 0.47 and 11.3 ± 0.81 mm, respectively. *β*-sitosterol (**2**) also showed antibacterial action against *E. coli*, *P. aeruginosa*, and *S. aureus* with IZ ranging from 7.0 ± 0.0–11 ± 0.0 mm. The highest IZ (11.0 ± 0.0 mm) was recorded against *S. aureus*. The antibacterial activities of the oil extract results showed that the oil extract showed promising antibacterial activity against all tested photogenes. *S. aureus* was the most inhibited bacteria with an IZ of 17.6 mm at 5 mg/mL concentration. The oil extract also displayed maximum antibacterial activity against *P. aeruginosa*, *E. coli*, and *S. pyogenes* with IZ of 14.3 ± 0.81, 15.0 ± 0.0, and 15.6 ± 0.47 mm, respectively (Table 6). The promising antibacterial potential of the oil extracts may be associated with the additive effect of the major and minor oil components. Fajrih et al. conducted a study on *B. gymnorrhiza* and identified 18.52% of palmitic acid which is reported to work synergistically with various active compounds; hence, it can increase its antibacterial activity [54]. Another study conducted by Tao et al. confirmed that tricosane displayed antibacterial activity against *Peltophorum fluorescens*, *E. coli*, *B. subtilis*, and *S. cerevisiae* with an IZ ranging from 8.20 to 15.97 mm at 20 mg/mL [55].

According to a study conducted by Octarya et al., eicosane has also been reported to have antibacterial activities [56]. The oil extract showed better antibacterial activity against all tested photogenes compared to the isolated compounds at the same concentration. The promising antibacterial potentials of the oil components may be linked with the synergistic or additive effects of its major and minor constituents [57]. The chemical diversity of essential oil components also may increase the possibility of existing constituents that can impair essential biochemical reactions needed for the survival of microorganisms [58]. The hydrophobicity of the fixed and essential oils may also significantly contribute to moving across the lipids of the cell membranes of bacteria and disrupt the cell wall structures [59].

#### 3.3.2. Radical Scavenging Activity

**Figure 3:**
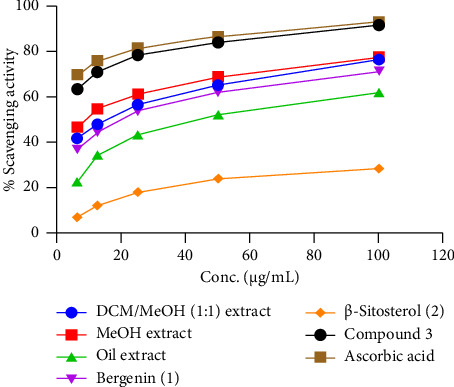
DPPH radical scavenging profile of crude extracts, oil extract, and compounds (**1**–**3**) from the roots of *W. uniflora*.

The radical scavenging potential of crude extracts, oil extract, and isolated compounds was evaluated following the DPPH assay using ascorbic acid as a standard. The radical scavenging activity result is given in Table 7 and Figure 3. The results revealed that compound **3** demonstrated promising scavenging of DPPH radical with a percentage inhibition of 91.8 ± 0.16% (IC_50_ value of 2.98 *μ*g/mL) compared to ascorbic acid with a percentage of inhibition of 93.2 ± 0.12% (IC_50_ value of 2.17 *μ*g/mL) at 100 *μ*g/mL (Table 7). The result of scavenging of DPPH radical of compound **3** showed in good agreement with the reported literature investigated by Utenova and co-workers (2007) [60]. DCM/MeOH (1:1) and MeOH extract also displayed significant scavenging of DPPH radical with a percentage inhibition of 76.8 ± 0.12± (IC_50_ value of 6.86 *μ*g/mL) and 77.8 ± 0.08% (IC_50_ value of 4.75 *μ*g/mL), respectively, at 100 *μ*g/mL (Table 7). The oil extract and bergenin (**1**) also exhibited promising scavenging of DPPH radical a percentage of inhibition of 62.0% and 71.4% ± 0.08%, respectively, at 100 *μ*g/mL. The lowest scavenging of DPPH radical was recorded by *β*-sitosterol (**2**) with a percentage of inhibition of 28.6% ± 0.12% (IC_50_ values of 24.14 *μ*g/mL) (Figure 3). Antioxidants have been investigated to show cell protection from free radicals causing oxidative cleavage which avoids aging and cancer, by interacting with free radicals, and thereby disrupting the oxidation process [61]. The promising antioxidant potential of the crude extracts and the isolated compounds may be due to the presence of polyhydroxy-aromatic groups that could donate hydrogen-bearing free radicals to stabilize DPPH free radicals. The observed promising scavenging potential of the crude extracts and isolated compounds suggests that the chemical constituents of the roots of *W. uniflora* may be used as a therapeutic agent for free radical–induced disorders.

#### 3.3.3. Molecular Docking Analysis

Humans alone have tens of thousands of proteins, and current experimental techniques make it very difficult to search through all of them in search of a query ligand [62]. Identifying the protein targets for a query ligand is an essential step in the drug discovery process. Molecular docking-based virtual screening techniques are crucial for identifying potential medication candidates from the large pool of structural data available [63]. In the ligand–receptor complex formation of a biochemical reaction, an enzyme whose elevated activity may associated with disease could be the target of interest; a drug that competitively inhibits the enzyme to lower its activity would be an interesting pharmacological precursor [64]. Human Prdx5 (PDB ID: 1HD2) is a thioredoxin reductase that reduces H_2_O_2_, alkyl hydroperoxides, and peroxynitrite. It is implicated in both target receptors for antioxidant compounds and signal transduction pathways [65, 66]. By upregulating Snail, Prdx5 overexpression increases carcinogenicity by encouraging the proliferation and invasiveness of gastric cancer cells [67].

**Figure 4:**
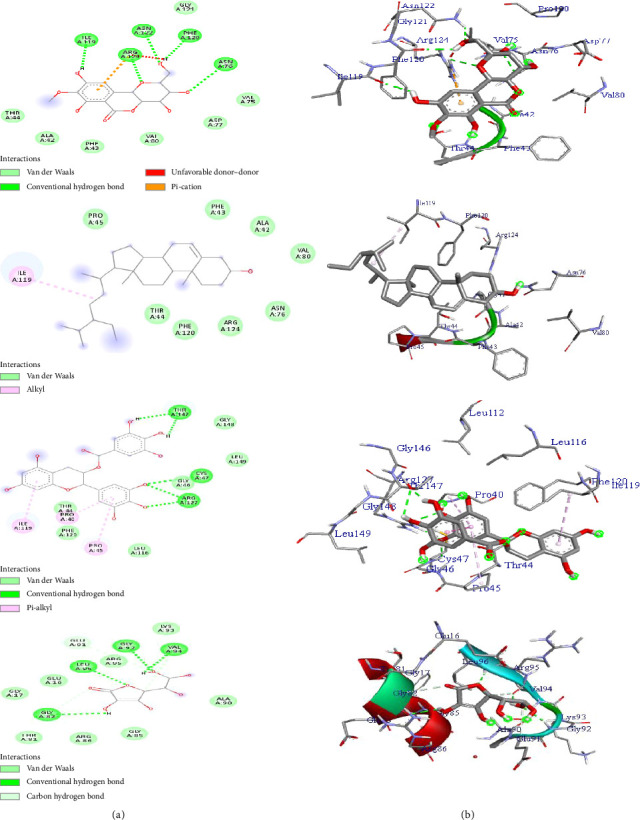
The 2D (a) and 3D (b) binding representation of compounds (**1**–**3**) and ascorbic acid toward peroxiredoxin 5 (PDB ID: 1DH2) receptor.

Human MPO is another antioxidant receptor that promotes oxidative stress in various inflammatory diseases [68]. MPO can catalyze the H_2_O_2_-mediated oxidation of chloride to the powerful oxidizing agent hypochlorous acid (HOCl), which leads to the oxidation (degradation) of biomolecules of pathogens in the phagosome. The initial product of MPO-H_2_O_2_-Cl^−^ system is the potent antimicrobial oxidant hypochlorous acid/hypochlorite (HOCl/OCl). However, recent evidence claims that excessive HOCl production can result in oxidative stress, tissue damage, initiation, and propagation of acute and chronic vascular inflammatory disease. HOCl can initiate modification reactions targeting lipids, DNA, and proteins through halogenation, nitration, and oxidative crosslinking [69, 70]. MPO-derived chlorinated compounds are specific markers for inflammation progression, which attracted considerable interest in the development of therapeutically useful MPO inhibitors and HOCl scavengers. Therefore, inhibition of the MPO target is an important mechanism for the treatment of free radical–induced disorders [69]. Topo II*α* is an important anticancer target enzyme involved in various stages of DNA replication, chromosome assembly, and segregation [71]. Topo II*α* expression has been used as a cancer cell marker because of its role in cell proliferation [72]. Topo II*α* catalytic inhibitors destroy cancer cells by preventing the formation of Topo II–DNA complex without increasing DNA cleavage, interfering with DNA binding, inhibiting cleavage of the DNA molecule, and binding to the ATP binding site [73]. Thus, the anticancer effect of topoisomerase II inhibitors is linked to the targeting of Topo II*α* [74]. Therefore, in this study, we predicted the binding orientation and affinity of the isolated compounds (**1**–**3**) toward human Prdx5 (PDB ID: 1HD2), human MPO (PDB ID: 1DNU), and Topo II*α* (PDB ID: 4FM9) receptors. The results of the molecular docking analysis of the isolated compounds (**1**–**3**) toward the target receptors are given in Tables 8, 9, and 10. All the analyzed compounds showed minimum binding energy (−5.2 to −6.3 kcal/mol) toward the human Prdx5 (PDB ID: 1HD2) target compared to ascorbic acid (−5.6 kcal/mol) (Table 8). The highest binding free energy was noted by compound **3** (−6.3 kcal/mol) followed by bergenin (**1**) (−6.2 kcal/mol). Compound **3** forms five hydrogen bond interactions through the active site of THR-A147 (2H-bonds), ARG-A127 (2H-bonds), and CYS-A47 amino acids. Moreover, residual amino acids PRO-A40, PRO-A45, ILE-A119 and GLY-A148, LEU-149, GLY-A46, LEU-A116, PHE-A120, and THR-A44 were involved through pi-alkyl and van der Waals force interactions, respectively (Table 8, Figure 4). The complex stabilization formed between bergenin (**1**) and PDB ID: 1HD2 receptor was established mainly by hydrogen bond interactions through the active sites of ILE-A119, ARG-A124, ASP-A122, PHE-A120, ASP-A76, and van der Waals force interactions through GLY-A121, THR-A44, ALA-A42, PHE-43, VAL-80, ASP-A77, and VAL-A75 amino acids (Figure 4).

*β*-sitosterol (**2**) exhibited minimum binding energy (−5.2 kcal/mole) toward the PDB ID 1HD2 receptor, compared to ascorbic acid (binding energy of −5.6 kcal/mol). As mapped in Table 8, residual amino acids PRO-A45, PHE-A43, ALA-A42, VAL-A80, ASP-A76, ARG-A124, PHE-A120, and THR-A44 contributed to the stabilization of the complex formed between *β*-sitosterol (**2**) and Prdx5 (PDB ID: 1HD2) receptor through van der Waals interactions (Table 8).

**Figure 5:**
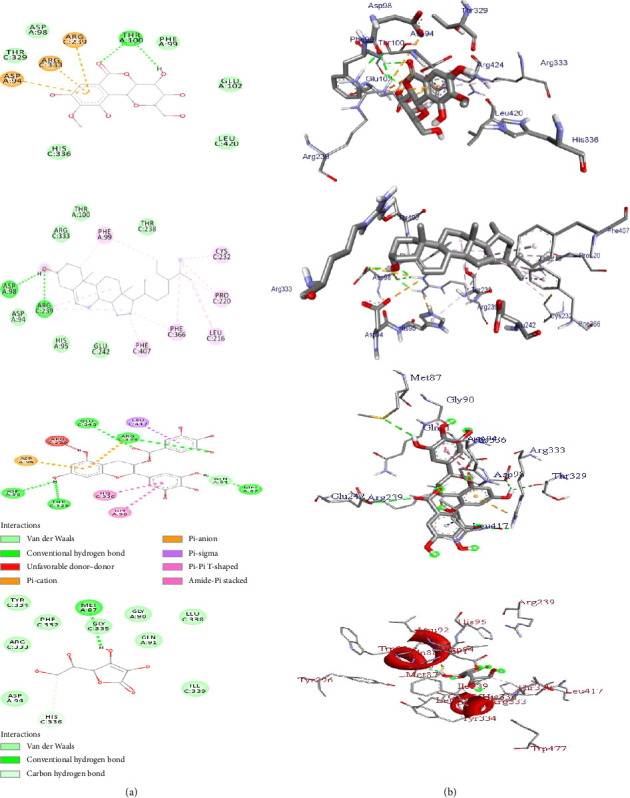
The 2D (a) and 3D (b) binding representation of compounds (**1**–**3**) and ascorbic acid toward human myeloperoxidase (PDB ID: 1DNU) receptor.

The molecular docking prediction of compounds (**1**–**3**) toward human MPO (PDB ID: 1DNU) receptor indicated that all the studied compounds displayed minimum binding affinity ranging from −8.0 to −10.7 kcal/mol compared to ascorbic acid (−5.7 kcal/mol) (Table 9). The best binding free energy (−10.7 kcal/mol) was noted by compound **3**. Bergenin (**1**) and *β*-sitosterol (**2**) also exhibited minimum binding affinity of −8.0 and −8.7 kcal/mol, respectively (Table 9). As mapped in Figure 5, hydrogen bond, van der Waals, and hydrophobic/*p*-alkyl interactions were involved through various amino acids in their corresponding ligand–protein complex. Compound **3** forms five hydrogen bond interactions in its ligand–protein complex toward the PDB: ID: 1DNU receptor through the active site of ASP-A98, THR-A-329, GLU-C242, ARG-C333, and MET-A87 amino acids (Table 9). *β*-sitosterol (**2**) interacts with PDB: ID 1DU receptor through hydrogen bond interactions via the active sites of ASP98 and ARG239 amino acid. Bergenin (**1**) also forms two hydrogen bonds via THR100 (2H-bonds) amino acid. Moreover, bergenin (**1**) exhibited electrostatic interaction in its ligand–protein complex through the active site of ARG-C333, ARG-C239, and ASP-A94 amino acids (Figure 5).

**Figure 6:**
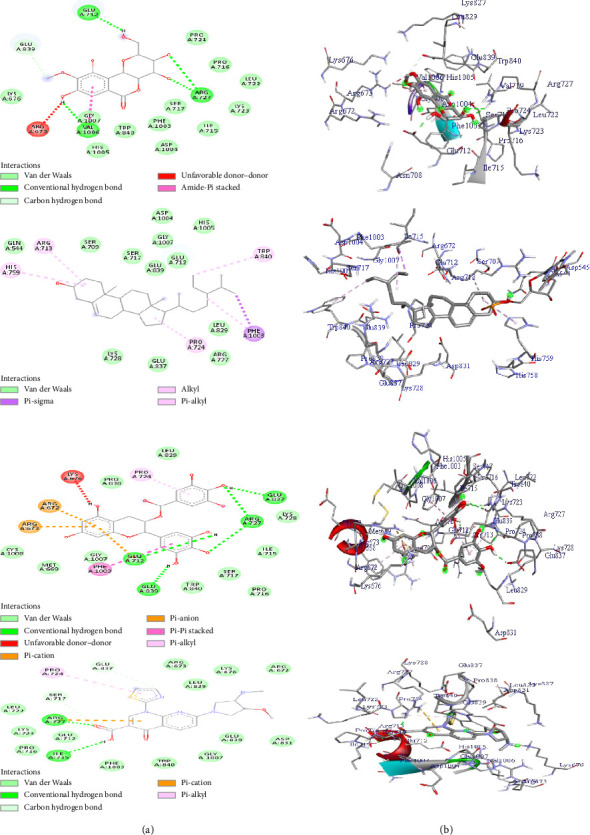
The 2D (a) and 3D (b) binding representation of compounds (**1**–**3**) against topoisomerase II*α* (PDB ID: 4FM9) receptor.

The molecular docking analysis of compounds (**1**–**3**) was also screened toward Topo II*α* (PDB ID: 4FM9) receptor, and the results are presented in Table 10 and Figure 6. The docking prediction demonstrated that all the analyzed compounds (**1**–**3**) showed minimum binding free energies ranging from −7.4 to −9.8 kcal/mol, compared to vosaroxin (−7.8 kcal/mol) (Table 10). The best binding free energy was recorded by compound **3** (−9.8 kcal/mol). Compound **3** forms three hydrogen bonds through the active sites of GLU-A837, GLU-A839, ARG-A727 (2H-bonds), and GLU-A712 amino acids (Figure 6). Residual amino acids LYS-A728, ILE-A715, SER-A717, TRP-A840, GLY-A1007, MET-A669, CYS-1008, PRO-A838, LEU-A829 and ARG-A673, ARG-A672, PHE-A1003, and PRO-A724 were involved in its ligand–protein complex through van der Waals and hydrophobic/pi-alkyl interactions, respectively (Figure 6). Bergenin (**1**) and *β*-sitosterol (**2**) exhibited binding free energy of −7.4 and 9.1 kcal/mol, respectively, compared to vosaroxin (−7.8 kcal/mol).

The complex formed between *β*-sitosterol (**2**) and the PDB ID: 4FM9 receptor was stabilized mainly by van der Waals interactions through GLN-A544, SER-A709, SER-A717, GLU-A839, GLY-A1003, ASP-A1004, HIS-A1005, LYS-A728, GLU-837, ARG-727, and hydrophobic/pi-alkyl interactions through the active site of HIS-A759, ARG-A713, PROA724, TRP-A840, and PHE-A1003 amino acids (Figure 6). Bergenin (**1**) forms four hydrogen bond interactions via GLU-A712, ARG-A727 (2H-bonds), and VAL-A1006 amino acids. Various residual amino acids were also involved through van der Waals and hydrophobic/pi-alkyl interactions in its ligand–protein complex (Table 10). The molecular docking analysis result revealed that all the studied compounds (**1**–**3**) exhibited minimum binding affinity toward the PDB ID: 4FM9 receptor and are expected to have anticancer potential by inhibiting Topo II*α* enzymes.

#### 3.3.4. Prediction of the Physicochemical and ADMET Parameter of the Isolated Compounds

The physicochemical and ADMET parameter of compounds (**1**–**3**) was evaluated using the Swiss ADME and PreADMET online tools following the standard guidelines described by Maliehe et al. [75]. The pharmacokinetic parameters of the isolated compounds were projected on the principle of Lipinski's rule of five [76, 77]. The physicochemical, ADMET, and toxicity profiles of the isolated compounds (**1**–**3**) are summarized in Table 11.

##### 3.3.4.1. Physicochemical Properties

The drug-likeness properties of the studied compounds were evaluated based on the principle of Lipinski's rule of five. According to Lipinski's rule, an orally active drug should meet at least four criteria of Lipinski's rule [78]. The Swiss ADME prediction result demonstrated that bergenin (**1**) and *β*-sitosterol (**2**) meet four and five criteria of Lipinski's rule of five, respectively, and are more likely administered orally. The computed TPSA value of *β*-sitosterol (**2**) was 20.23 Å and is well below the limit of 140 Å, indicating its optimal permeability and well below the anticancer target, vosaroxin (136.13 Å) (Table 11). The examined compounds (**1**–**3**) all had lipophilicity (iLogP) values ranging from 1.32–4.79, indicating their ideal lipophilicity [79]. The molecular weight of a drug candidate is also an important parameter in the drug's mechanism of action. The smaller the molecular weight (MW ≤ 500 g/mole), the lighter molecules can easily be absorbed and diffused in the body, whereas the poorer drug uptake and bioavailability are associated with greater molecular weights (MW > 500 g/mole) [80]. The Swiss ADME estimation result also showed that compounds (**1**–**3**) have acceptable molecular weights (MW ≤ 500 g/mole), which indicated their capacity to be swiftly absorbed and transported due to their compliance with Lipinski's rule of five concerning MW (MW ≤ 500 g/mole). The conformational stability of a drug candidate is related to the number of rotatable bond (NRB) values. Ligands to have good bioavailability have ≤ 10 rotatable bonds [81]. The NHA and NHD of bergenin (**1**) and *β*-sitosterol (**2**) were determined to be within Lipinski's limit range, suggesting that the compounds can be readily absorbed or permeable from the gastrointestinal system.

##### 3.3.4.2. ADMET Properties

SwissADMET online tool was used to predict the in silico pharmacokinetics properties (ADMET) of isolated compounds (**1**–**3**). Skin permeability (Kp), a crucial factor in determining a molecule's potential drug-likeness, refers to a molecule's capacity to pass through the skin's outer layer. The less skin permeant the molecule, the more negative the log Kp value [82]. All of the examined compounds (**1**–**3**) had log Kp values that fall within the permitted range of −8.33 to −2.20 (cm/s), indicated limited skin permeability, and are within the range of the anticancer agent, vosaroxin (−9.07 cm/s) (Table 10). The lowest skin permeate was recorded by bergenin (**1)** with log Kp values of −8.33 cm/s. The Swiss ADME screening indicated that none of the tested compounds (**1**–**3**) are P-gp substrates or inhibitors of the majority of the chosen cytochromes (CYP). Only CYP3A4 enzyme showed inhibition by compound **3** (Table 11). Bergenin (**1**) showed high GI absorption, indicating it can readily permeability across the gastrointestinal tract. All compounds (**1**–**3**) also showed that none of the compounds exhibited BBB permeability. Thus, the compounds are expected to be anticipated to have a high level of CNS safety [83].

##### 3.3.4.3. Toxicity Profile

The Pro Tox II screening analysis indicated that all compounds (**1**–**3**) exhibited median fatal dose (LD_50_) values ranging from 547 to 10000 mg/kg (Table 11). The results obtained from the acute toxicity screening indicated that bergenin (**1**) did not exhibit acute toxicity, as shown by the toxicity class categorization of 6 (nontoxic). Compound **3** exhibited toxicity class classification 4 (harmful if swallowed). It was projected that none of the isolated compounds would be cytotoxic, mutagenic, or hepatotoxicity. Bergenin (**1**) and compound **3** also did not show immunotoxicity (Table 11). The ADMET prediction results indicated that the isolated compounds may be a promising medication candidate.

## 4. Conclusion

In the present study, silica gel column chromatography separation of CH_2_Cl_2_/MeOH (1:1) and MeOH roots extracts of *W. uniflora* afforded three compounds (**1**–**3**), reported herein for the first time from the species. Hydrodistillation extraction of roots of *W. uniflora* led to the identification of five compounds, of which palmitic acid (34.9%) was the major constituent. The in vitro antibacterial activities result showed that the oil extract demonstrated promising antibacterial activities against all tested bacterial strains with IZ ranges of 14.3 ± 0.81 to 17.6 ± 0.47 mm at 5 mg/mL. CH_2_Cl_2_/MeOH (1:1) and MeOH crude extracts and compounds (**1**–**3**) showed low-to-moderate antibacterial activity (9.6 ± 0.47–13.6 ± 0.47 mm), compared to ciprofloxacin, but undeniably important. The promising antibacterial activity of the oil extracts may be associated with the additive effects of the oil constituents and their hydrophobicity to easily move across the bacteria's cell membrane and disrupt the bacteria's cell wall. On the other hand, the crude extracts and compounds (**1** and **3**) demonstrated promising scavenging of DPPH radical with a percentage of inhibition ranged from 76.8 ± 0.12 to 91.8 ± 0.16%. The promising antioxidant potential of the compounds may be due to the presence of hydroxy-aromatic groups that could donate hydrogen-bearing free radicals to stabilize DPPH free radicals. The molecular docking prediction results demonstrated that all compounds (**1**–**3**) exhibited minimum binding energy toward PDB ID: 1HD2 (−5.2 to −6.3 kcal/mol) and PDB ID: 1DNU (−8.0 to −10.7 kcal/mol) receptors. Toward PDB ID: 4FM9 receptor, the best binding free energy was noted by compound **3** (−9.8 kcal/mole), compared to vosaroxin (−7.8 kcal/mole). The drug-likeness analysis results revealed that bergenin (**1**) and *β*-sitosterol (**2**) meet four and five rules out of Lipinski's rule of five, and are more likely to be taken oral medications. The in vitro antibacterial and antioxidant activity results supported with in silico analysis demonstrated that the chemical constitutions of the roots of *W. uniflora* have therapeutic potency in treating diseases associated with pathogenic bacteria and free radical–inducing disorders. Further comprehensive investigations including in vivo activity tests could be conducted for a conclusive decision on the potential medication of the plant for formulation and therapeutic applications.

## Figures and Tables

**Table 1:** ^1^H and ^13^C NMR (400 MHz, in CD_3_OD) of bergenin (**1**) (*δ* in ppm).

C/H	Bergenin (1) (δ in ppm)					Literature [34]	
	^1^H NMR	^13^C NMR	^1^H-^1^H COSY	HSQC	^1^H-^13^C HMBC	^1^H NMR	^13^C NMR
2	3.68 (1H, m)	82.9	H_2_ ⟶ H_3_	H_2_ ⟶ C_2_	H_2_ ⟶ C_3,4,10b,11_	3.81 (1H, dd, *J* = 9.0, 5.7)	83.1
3	3.44 (1H, dd, *J* = 9.5, 8.7)	71.8	H_3_ ⟶ H_2,4_	H_3_ ⟶ C_3_	H_3_ ⟶ C_2,4,11_	3.42 (1H, dd, *J* = 9.2, 9.0 Hz)	71.9
4	3.81 (1H, dd, *J* = 9.5, 8.7)	75.6	H_4_ ⟶ H_3,4a_	H_4_ ⟶ C_4_	H_4_ ⟶ C_3,4a_	4.02 (dd, 10.0, 9.2 1H)	75.7
4a	4.05 (1H, dd, *J* = 10.4, 9.5)	81.3	H_4a_ ⟶ H_4,_H_10b_	H_4a_ ⟶ C_4a_	H_4a_ ⟶ C_4,10a,10b_	4.10 (1H, dd, *J* = 10.4, 10.1)	81.5
6	—	165.7	—	—			165.8
6a	—	119.3	—	—		—	119.5
7	7.05 (1H, s)	111.0	—	—	H_7_ ⟶ C_6,6a,8,9,10a_	7.08 (s 1H)	111.1
8	—	152.2	—	—		—	152.4
9	—	142.2	—	—			142.3
10		149.3	—	—			149.5
10a		117.2	—	—			117.3
10b	4.94 (1H, d, *J* = 10.4)	74.2	H_10b_ ⟶ H_4a_	H_10b_ ⟶ C_10b_	H_10b_ ⟶ C_2,4,4a,9,10,10b_	4.95 (d, *J* = 10.4 1H)	74.3
11	4.02 (1H, m); 3.72 (1H, m)	62.6	H_11_ ⟶ H_2_	H_11*α*,*β*_ ⟶ C_11_	H_11_ ⟶ C_3_	3.68 (2H, m, H-11)	62.7
12	3.89 (3H, s)	60.8	—	H_12_ ⟶ C_12_	H_12_ ⟶ C_8, 9_,_10_	3.95 (3H, s)	60.9

**Table 2:** ^1^H and ^13^C NMR (400 MHz, in CDCl_3_) of *β*-sitosterol (**2**) (*δ* in ppm).

Position C/H	β-sitosterol (2)		Literature [44]	
	^1^H NMR	^13^C NMR	^1^H NMR	^13^C NMR
1	1.84 (m)	37.2	1.85 (m)	37.3
2	1.95 (m)	31.5	1.95 (m)	32.0
3	3.53 (tdd)	71.7	3.52 (m)	71.9
4	2.25 (d, *J* = 7)	42.3	2.24 (dd, *J* = 1.44; 1.06) and 2.38, t)	42.4
5	—	140.7	—	140.8
6	5.36 (t, 1H, *J* = 6.4 Hz)	121.7	5.35	121.8
				
7	2.02 (dd)	31.9	1.99 (m)	31.7
8	1.98 (m)	31.9	2.00 (m)	32.0
9	0.95 (m)	50.1	0.94 (m)	50.2
10	—	36.4	—	36.6
11	1.02 (m)	21.0	1.02 (m)	21.2
12	1.14 (m)	39.7	1.16 (m)	39.8
13	—	42.1	—	42.3
14	1.00 (m)	56.7	1.00 (m)	56.9
15	1.52 (m)	24.3	1.06 (m) and 1.58 (m)	24.4
16	1.09 (m)	28.2	1.09 (m)	28.3
17	1.11 (m)	56.0	1.12 (m)	56.0
18	0.70 (3H, s)	11.8	0.85 (s)	12.1
19	1.01 (s)	19.4	0.82 (s)	19.5
20	1.16 (m)	36.1	1.16 (m)	36.3
21	1.02 (d, 3H)	18.7	1.03 (d, *J* = 7.2 Hz, 3H)	18.9
22	1.33 (m)	33.9	1.33 (m)	34.0
23	1.16 (m)	26.0	1.16 (m)	26.2
24	0.93 (m)	45.7	0.94 (m)	45.9
25	1.66 (m)	29.1	1.66 (m)	29.2
26	0.87 (d, 3H)	19.8	0.83 (d, *J* = 11 Hz)	19.3
27	0.85 (d, 3H)	19.0	0.84 (d)	19.1
28	0.84 (d, 3H)	23.0	1.25 (m)	23.2
29	0.82 (t, 3H)	11.9	0.85 (t)	12.0

**Table 3:** ^1^H and ^13^C NMR of epigallocatechin 3-gallate (**3**) (400 MHz, in DMSO − *d*_6_) (*δ* in ppm).

Position	Epigallocatechin 3-gallate (3)		Literature [46]	
	^1^H NMR	^13^C NMR	^1^H NMR	^13^CNMR
2	5.35 (d)	77.9	5.56 (d)	78.6
3	4.94 (dd)	68.5	4.92 (s)	69.9
4	2.65–2.92 (2H, m)	26.1	3.03–2.92 (m, H)	26.8
5	—	156.9	—	156.7
6	5.93 (2H, s)	95.9	5.94 (s)	96.5
7	—	156.9	—	157.8
8	5.83 (2H, s)	94.7	(5.94, s)	95.9
9	—	156	—	157.8
10	—	97.8	—	99.4
1′	—	129.0	—	130.8
2′&6′	6.40 (2H, s)	105.9	6.49 (s)	106.9
3′&5′	—	146.0	—	146.7
4′	—	132.7	—	133.8
1″	—	119.7		121.4
2″&6″	6.81 (2H, s)	109.0	6.94 (s, 2H)	110.2
3″&5″		145.8	—	146.7
4″		139.0		139.8
7″		165.7	—	167.7

**Table 4:** Essential and fixed oils identified from the root of *W. uniflora*.

Compound name	Molecular formula	Retention time (*t*_*R*_)	Retention index (RI)	Relative percentage (%)
Cyclopropene, 2,3-dimethyl-3-phenyl	C_11_H_12_	14.84	1140	13.37
(1R,7S,E)-7-Isopropyl-4,10-dimethylenecyclodec5-enol	C_15_H_24_O	23.84	1695	20.81
Palmitic acid	C_16_H_32_O_2_	24.04	1968	34.9
Tricosane	C_23_H_48_	27.09	2300	12.67
Eicosane	C_20_H_42_	28.72	2000	18.22
Total				99.97

**Table 5:** Antibacterial activities (mean ± SD, in mm) of CH_2_Cl_2_/MeOH (1:1) and MeOH root extracts of *W. uniflora*.

Bacterial stains	Conc. (mg/mL)	CH_2_Cl_2_/MeOH (1:1) extract	MeOH extract	Cipro (30 μg/disk)
*E. coli*	200	11.6 ± 0.81	13.6 ± 0.47	27.0 ± 0.0
	100	8.3 ± 0.81	11.6 ± 0.81	
	50	7 ± 0.0	9.3 ± 0.57	

*P. aeruginosa*	200	10.0 ± 0.0	9.0 ± 0.0	29.0 ± 0.0
	100	9.3 ± 0.81	8.3 ± 0.81	
	50	8.0 ± 0.0	7.0 ± 0.0	

*S. aureus*	200	NI	11.6 ± 0.47	31.0 ± 0.0
	100	NI	9.3 ± 0.81	
	50	NI	8.0 ± 0.0	

*S. pyogenes*	200	NI	10.6 ± 0.47	30.0 ± 0.0
	100	NI	8.6 ± 0.47	
	50	NI	7.0 ± 0.0	

Abbreviation: NI, no inhibition.

**Table 6:** Antibacterial activity (mean ± SD) of oil extract and compounds (**1**–**3**) isolated from roots of *W. uniflora*.

Bacterial strain	Conc. (mg/mL)	Isolated compounds			Oil extract	Cipro (30 μg/disk)
		1	2	3		
*E. coli*	5	11.6 ± 0.47	10.6 ± 0.47	11.3 ± 0.81	15.0 ± 0.0	27.0 ± 0.0
	2.5	10.0 ± 0.0	8.6 ± 0.0	8.0 ± 0.0	11.0 ± 0.0	
	1.25	8.0 ± 0.0	7.3 ± 0.04	7.0 ± 0.0	10.3 ± 0.04	

*P. aeruginosa*	5	9.3 ± 0.81	9.3 ± 0.81	8.3 ± 0.81	14.3 ± 0.08	29.0 ± 0.0
	2.5	8.6 ± 0.47	7.0 ± 0.0	7.6 ± 0.47	12.0 ± 0.0	
	1.25	8.0 ± 0.0	7.0 ± 0.00	7.3 ± 0.81	11.6 ± 0.47	

*S. aureus*	5	10.6 ± 0.047	11.0 ± 0.0	NI	17.6 ± 0.47	31.0 ± 0.0
	2.5	9.0 ± 0.0	8.3 ± 0.81	NI	14.3 ± 0.81	
	1.25	7.3 ± 0.81	7.6 ± 0.47	NI	11.0 ± 0.0	

*S. pyogenes*	5	8.0 ± 0.0	NI	7.0 ± 0.0	15.6 ± 0.47	30.0 ± 0.0
	2.5	7.3 ± 0.81	NI	NI	13.6 ± 0.47	
	1.25	7.0 ± 0.0	NI	NI	11.3 ± 0.81	

Abbreviation: NI, no inhibition.

**Table 7:** DPPH scavenging potential of (mean ± SD) crude extracts, oil extract, and compounds (**1**–**3**) from the roots of *W. uniflora*.

Conc. (μg/mL)	Crude extracts		Oil extract	Isolated compounds			Ascorbic acid
	DMC/MeOH (1:1)	MeOH (1:1)		1	2	3	
100	76.8 ± 0.12	77.8 ± 0.08	62.0 ± 0.0	71.4 ± 0.08	28.6 ± 0.12	91.8 ± 0.16	93.2 ± 0.04
50	65.2 ± 0.08	69.0 ± 0.0	52.2 ± 0.16	62.2 ± 0.04	24.2 ± 0.08	84.0 ± 0.0	87.0 ± 0.0
25	57.0 ± 0.0	61.4 ± 0.08	43.6 ± 0.04	54.0 ± 0.16	18.0 ± 0.12	78.6 ± 0.02	81.8 ± 0.08
12.5	48.2 ± 0.16	48.2 ± 0.16	35.5 ± 0.16	44.6 ± 0.0	12.3 ± 0.08	71.2 ± 0.12	76.0 ± 0.09
6.25	41.8 ± 0.12	46.8 ± 0.14	22.8 ± 0.08	36.8 ± 0.02	7.0 ± 0.16	63.8 ± 0.08	70.2 ± 0.04
IC_50_ (μg/mL)	6.86	4.75	13.15	7.15	24.14	2.98	2.17

**Table 8:** Binding interaction profile of compounds (**1**–**3**) toward human peroxiredoxin 5 (PDB ID: 1HD2).

Compound	Affinity (kcal/mol)	H-bond	Residual interactions	
			Hydrophobic/pi-alkyl	Van der Waals force
1	−6.2	ILE-A119, ARG-A124, ASP-A122, PHE-A120, ASP-A76	—	GLY-A121, THR-A44, ALA-A42, PHE-43, VAL-80, ASP-A77, VAL-A75
2	−5.2	—	ILE-A119	PRO-A45, PHE-A43, ALA-A42, VAL-A80, ASP-A76, ARG-A124, PHE-A120, THR-A44
3	−6.3	THR-A147 (2H-bonds), ARG-A127(2H-bonds), CYS-A47	PRO-A40, PRO-A45, ILE-A119	GLY-A148, LEU-149, GLY-A46, LEU-A116, PHE-A120, THR-A44
Ascorbic acid	−5.6	LEU-A96, GLT-A92, VAL-A94, GLY-A82,	LYS-93, ALA-A90, GLY-A85, ARG-A80,THR-A81, GLY-A17, GLU-A16	GLU-A91

**Table 9:** Binding profile of compounds (**1**–**3**) and ascorbic acid toward human myeloperoxidase (PDB ID 1DNU) receptor.

Compound	Affinity (kcal/mol)	H-bond	Residual interaction	
			Hydrophobic/pi-cation, pi-anion, pi-alkyl interactions	Van der Waals force
1	−8.0	THR100	—	GLU102, LEU420, PHE 99, ASP98, THR329, HIS339, ARG239, ARG333
2	−8.7	ASP98, ARG239	ASP94, HIS95, GLU242, ARG333, THR100, THR 238	PHE99, PHE366, CYS239, PRO220, LEU216
**3**	−10.7	ASP-A98, THR-A-329, GLU-C242, ARG-C333,MET-A87	HISC336, GLY-A90, ASP-A94, LEU-C417	—
Ascorbic acid	−5.7	MET-A87	HIS-C336	TYR-C334, ARG-C333, PHE-C332, GLY-C 335, GLY-A90, GLN-A91, LEU-C338, ILE-C339, ASP A94

**Table 10:** Binding profile of compounds (**1**–**3**) toward Topoisomerase II*α* (PDB ID: 4FM9).

Compound	Affinity (kcal/mol)	H-bond	Residual interactions	
			Hydrophobic/Pi-Cation, pi/alkyl, pi-anion	Van der Waals
1	−7.4	GLU-A712, ARG-A727(2H-bonds), VAL-A1006	GLU-A839, GLY-A1007	LYS-A670, GLY-A1007, HIS-A1005, TRP-A840, PHE–A1003, ASP-A1004, ILE-A715, LYS-A723 LEU-A722, PRO-A716, PRO-A724
2	−9.1	—	HIS-A759, ARG-A713, PROA724, TRP-A840,PHE-A103	GLN-A544, SER-A709, SER-A717,GLU-A839, GLY-A1003, ASP-A1004, HIS-A1005, LYS-A728, GLU-837, ARG-727
3	−9.8	GLU-A837, GLU-A839, ARG-A727 (2H-bonds), GLU-A712	ARG-A673, ARG-A672, PHE-A1003, PRO-A724	LYS-A728, ILE-A715, SER-A717, TRP-A840, GLY-A1007, MET-A669, CYS-1008, PRO-A838, LEU-A829
Vosaroxin	−7.8	ARG-A727, ILE-715	PRO-A724, SER-A717, GLU-837	ARG-A673, LEU-A829, LYS-A676, ARG-A672, ASP-A831, GLU-839, GLY-A1007, TRP-A716, LYS-A723, LEU-A722

**Table 11:** Pharmacokinetics, ADMET, and toxicity profile of isolated compounds (**1**–**3**).

Parameter		Bergenin (1)	*β*-sitosterol (2)	Compound 3	Vosaroxin
Formula		C_14_H_16_O_9_	C_29_H_50_O	C_23_H_17_O_10_	C_18_H_21_N_5_O_4_S
Molecular weight (g/mole)		328.27	414.71	441.40	403.46 g/mol
No. of hydrogen donors		5	1	7	2
No. of hydrogen acceptors		9	1	11	7
No. of rotatable bonds		2	6	5	5
TPSA (Å^2^)		145.91	20.23	186.37	136.13
LogP (iLogP)		1.32	4.79	1.53	1.97
Lipinski's rule of five violation		1	0	3	0
Log Kp (cm/s)		−8.33	−2.20	−7.36	−9.07
Gastrointestinal absorption		High	Low	High	High
Blood–brain barrier (BBB)		No	No	No	No
Inhibitor interaction	P-gp Substrate	No	No	Yes	Yes
	CYP1A2	No	No	Yes	No
	CYP2C19	No	No	No	No
	CYP2C9	No	No	No	No
	CYP2D6	No	No	No	No
	CYP2D6	No	No	No	No
Toxicity	Hepato	No	No	No	No
	Carcino	No	No	No	No
	Immuno	No	Yes	No	No
	Mutagen	No	No	No	No
	Cyto	No	No	No	No
LD_50_ (mg/kg)		10,000	5000	1000	2000
Toxicity class		6	5	4	4

## Data Availability

The NMR spectra used to establish the structures of the isolated compounds in this work are included as Supporting Information.

## Supporting Information

Supporting Information 1A: ^1^H NMR spectrum (in CD_3_OD) of bergenin (**1**). Supporting Information 1B: ^13^C NMR spectrum (in CD_3_OD) of bergenin (**1**). Supporting Information 1C: DEPT-135 spectrum of (in CD_3_OD) of bergenin (**1**). Supporting Information 1D: HSQC spectrum (in CD_3_OD) of bergenin (**1**). Supporting Information 1E: ^1^H-^1^H COSY spectrum (in CD_3_OD) of bergenin (**1**). Supporting Information 1F: HMBC spectrum (in CD_3_OD) spectrum of bergenin (**1**). Supporting Information 2A: ^1^H NMR spectrum (in CDCl_3_) of *β*-sitosterol (**2**). Supporting Information 2B: ^13^C NMR spectrum (in CDCl_3_) of *β*-sitosterol (**2**). Supporting Information 2C: DEPT-135 spectrum (in CDCl_3_) of *β*-sitosterol (**2**). Supporting Information 3A: ^1^H NMR spectrum (in DMSO − *d*_6_) of compound **3**. Supporting Information 3B: ^13^C NMR spectrum (in DMSO − *d*_6_) of compound **3**. Supporting Information 3C: DEPT-135 spectrum (in DMSO − *d*_6_) of compound **3**.
