# Supplementary material for: Evaluation of the Antibacterial and Antioxidant Properties of Chemical Constituents of the Roots of Woodfordia uniflora: An Integrated Approach of Experimental and Computational Study
Source: Biochem Res Int. 2024 Dec 3;2024:1322756. doi: 10.1155/bri/1322756 (PMC11631344; doi:10.1155/bri/1322756)
Supplement: Supporting Information — Additional supporting information can be found online in the Supporting Information section. [file 1322756.f1.docx]

**Supplementary Information**

**Bihon Abera^1^, Negera Abdissa^2^, Milkyas Endale^2*^, Yadessa Melaku^1^, Kebede Shenkute^1^, Urgessa Ensermu ^3^, Mo Hunsen ^4^, Daniel Rentsch^5^ and Rajalakshmanan Eswaramoorthy^6^**

^1^Department of Applied Chemistry, School of Applied Natural Science, Adama Science and Technology University, P.O.Box 1888, Adama, Ethiopia.

^2^Traditional and Modern Medicine Research and Development Directorate, Armauer Hansen Research Institute, Post Box 1005, Addis Ababa, Ethiopia.

^3^Department of Applied Biology, School of Applied Natural Science, Adama Science and Technology University, P.O.Box 1888, Adama, Ethiopia.

^4^ Department of Chemistry, Kenyon College, Gambier, OH 43022, USA.

^5^Laboratory for Functional Polymers, Empa, Swiss Federal Laboratories for Materials Scienceand Technology, Überlandstrasse 129, 8600 Dübendorf, Switzerland.

^6^Department of Biomaterials, Saveetha Dental College and Hospitals, Saveetha Institute of Medical and Technical Science, Saveetha University, Chennai, India.

Correspondence: milkyasendale@yahoo.com

Supplementary Information 1A: ^1^H NMR (CD_3_OD) of Bergenin (**1)**.

Supplementary Information 1B: ^13^C NMR (CD_3_OD) of Bergenin (**1)**.

Supplementary Information 1C: DEPT-135 (CD_3_OD) of Bergenin (**1**).

Supplementary Information 1D: HSQC (CD_3_OD) spectrum of Bergenin (**1**).

Supplementary Information 1E: ^1^H-^1^H COSY (CD_3_OD) spectrum of Bergenin (**1**).

Supplementary Information 1F: ^1^ HMBC (CD_3_OD) spectrum of Bergenin (**1**).

Supplementary Information 2A: ^1^H NMR (CDCl_3_) of β-sitosterol (**2**)

Supplementary Information 2B: ^13^C NMR (CDCl_3_) of β-sitosterol (**2**)

Supplementary Information 2C: DEPT-135 (CDCl_3_) of β-sitosterol (**2**)

Supplementary Information 3A: ^1^H NMR (DMSO-*d*_6_) of Compound **3**.

Supplementary Information 3B: ^13^C NMR (DMSO-*d*_6_) of Compound **3**.

Supplementary Information 3C: DEPT-135 (DMSO-*d*_6_ ) of Compound **3**.
